# Management practices of primary postpartum hemorrhage among traditional birth attendants: a qualitative survey, Iganga, Uganda

**DOI:** 10.4314/ahs.v25i4.14

**Published:** 2025-12

**Authors:** Sharon Namasambi, Kizito Omona

**Affiliations:** 1 Research Coordination Centre, Mildmay Uganda, Kampala; 2 Faculty of Health Sciences, Uganda Martyrs University, Kampala

**Keywords:** Postpartum hemorrhage, Traditional Birth Attendants, Skilled Birth Attendants, Management Practices, Uganda

## Abstract

**Objectives:**

Postpartum hemorrhage is a significant barrier to the achievement of Sustainable development goal 3.1, given that it is associated with more than a quarter of all maternal mortality cases. Traditional birth attendants (TBAs), who are outlawed in many countries, including Uganda inclusive, attend to about 33% of all births globally. The objective of this study was to explore the management practices of primary postpartum hemorrhage among traditional birth attendants in Iganga district.

**Methods:**

The study used a case phenomenological qualitative exploratory design, targeting traditional birth attendants. Snowballing was used to select participants and engage them in-depth interviews. Data collected was analyzed thematically, with the induction approach.

**Results:**

It was found that TBAs carried out PPH assessment using three methods; quantification of blood lost using household cups, vital assessment and visual assessment. Visual assessment was the most dominant. Postpartum hemorrhage resuscitation practices mainly rotated around the use of warm water. They did not carry resuscitation. The treatment interventions used were: use of herbal medicine and referral of severe cases to hospital.

**Conclusion:**

TBAs in Iganga district visually assess postpartum hemorrhage but they do not conduct resuscitation procedures. They use herbal uterotonics in the treatment of postpartum hemorrhage alongside with referral to hospital.

## Introduction and background

Reductions in maternal mortality that have been registered over the past two decades are undeniably significant, having reduced by 38%, as of the year 2019^1^,^2^. This reduction only represents an annual rate of 2.9% and yet the reduction rate required to achieve sustainable development goal 3.1 (SDG 3.1) by 2030 is 6.4%^3^. More than 30% of the deaths occur in the postpartum period^4^,^5^ and are anteceded by intrapartum complications. The most notable among the postpartum complications, is postpartum hemorrhage (PPH)^6^–^8^, that is associated with more than 25% of all maternal deaths^6^. Postpartum hemorrhage is primarily specified by vaginal bleeding that exceeds 500 ml after vaginal delivery or cesarean delivery. It is one of the main causes of maternal near misses^7^,^9^. It predisposes mothers to acute cyanosis, shock, and non-responsive oliguria and hematological disorders^10^,^11^. All the aforementioned clinical indicators, being fatal, further buttress PPH as a mortality risk incrementing obstetric complications^12^. With progression to severity, interventions including use of balloon tamponade and the implementation of compression sutures are done^13^–^15^. Although usually conservatively done, the interventions are known to increase risk of experiencing spontaneous abortions in future pregnancies and risk of cesarean birhs at term^14^. This is in addition to the fact that the aforementioned interventions increase the risk of hysterectomies^16^–^18^, laparotomy and sepsis and yet, in most cases, the interventions are unavoidable, given that mothers with severe PPH do not respond to first line treatment^19^.

Whereas PPH is preventable and manageable with Skilled Birth Attendance (SBA), skilled births attendants are in short supply in Africa, where 80% of all maternal deaths occur. Each year, millions of births still occur without any assistance from a skilled attendant despite recent progress^20^. More than a third of all child births are attended to by unskilled birth attendants, among whom are Traditional Birth Attendants (TBAs)^21^,^22^.

Uganda was projected to have 75% of all baby deliveries performed by skilled birth attendants by end 2020 but Traditional Birth Attendances (TBAs) have made meeting this target very hard to achieve. Despite having no formal training, TBAs have provided maternity care during and after pregnancy and childbirth. They are still very active in developing countries although not recognized as medical practitioners. They command a high societal standing and many families seek TBAs as health care providers^23^,^24^. This study, thus, explored the management practices (assessment, resuscitation and referral practices) of post-partum hemorrhage among traditional birth attendants in Iganga, a rural district in Eastern Uganda.

## Materials and methods

### Study design and area

We used a phenomenological qualitative exploratory design^25^, and conducted interviews among traditional birth attendants in Iganga district. Iganga is one of the districts that have persistently had skilled birth attendance rates not exceeding 65% to 70%.

### Study population and sample size

We targeted the traditional birth attendants in Iganga district who had been active within the past three years prior to the study, and had conducted deliveries of more than two mothers with postpartum hemorrhage. The number of TBAs who were sampled was determined using the data saturation principle. Data saturation was noticed at the 7th in-depth interview and confirmed by conducting one subsequent interview, which made the sample size to be eight (8) TBAs.

### Sampling procedures

In liaison with local Non-governmental Organizations (NGOs) who had earlier on conducted a study among TBAs in the district, we were able to identify and purposively sample the first TBA and interviewed her and then requested her to provide information related to the location of other TBAs. Once information was obtained from a given TBA, we did snowballing in order to locate all the subsequent TBAs. This process was done until saturation was reached.

### Data collection methods and tools

We used in-depth interviews as the only data collection method. The use of in-depth interviews was viable enough to extract the data required for this study. All the interviews were moderated by the principal investigator, with the help of an assistant whose role was to take note and mane voice recording. Each interview took between one hour to one and half hours. The data collection tool used was designed, with cognizance of all the three objectives that the study had. Each of the questions was accompanied with probe options to ensure that all the possible opinions were exhausted.

### Operational Definitions

In this study, the following definitions have been defined as follows;

1) Postpartum period - The postpartum period (puerperium) is defined as the 6-week period of time beginning immediately after child birth, during which the reproductive organs and maternal physiology return toward the pre-pregnant state^26^(p2).
2) Primary versus secondary Postpartum haemorrhage - Primary postpartum hemorrhage is bleeding that occurs in the first 24 hours after delivery, while secondary post-partum hemorrhage is characterized as bleeding that occurs 24 hours to 12 weeks postpartum^27^.
3) Traditional Birth Attendants - A Traditional birth attendant (TBA) is a person who assists a mother during childbirth and who initially acquired her skills by delivering babies herself or through apprenticeship to other traditional birth attendants^28^.
4) Skilled Birth Attendants - A skilled birth attendant (SBA) is an accredited health professional, such as a midwife, a doctor or a nurse, who has been educated and trained to proficiency in the skills needed to manage normal pregnancies, childbirth and the immediate postnatal period and in the identification, management and referral of women and neonates for complications^29^.
5) Management Practices – In this study it is referred to management activities which relate to assessment, resuscitation and referral of post-patum hemorrhage mothers by Traditional Birth Attendants^18^.

### Data Management and Analysis

We played the voice records, for purposes of ensuring that none of them was broken, and to ensure that they were complete. Following the verification of the completeness of the audio records, counter verification was also conducted with the notes that had been taken for purposes of ensuring complete set of data prior to transcription. The analysis of data was conducted using the thematic analysis approach^30^. Analysis was done through a six-phase process, that started with the familiarization with the data in Phase 1, generation of initial codes in Phase 2, search for themes in phase 3, review of themes in phase 4 and definition and naming of themes in phase 5 and 6 in that respective order.

## Ethical considerations

All the required administrative clearance was sought and granted by the responsible authorities in Iganga district and final Institution Review Board (IRB) approval was granted by Mildmay Uganda Research Ethics Committee on 20th June, 2019 under IRB approval reference number (REC REF: 1405-2019). Participation in the study was voluntary and signed informed consents were duly sought. The principle of confidentially and anonymity was observed.

## Results

### Socio demographic characteristics

The majority of the TBAs sampled was aged above 50 years (5/8), and were married. Half of the traditional birth attendants who participated in the study had been in the practice for more than 30 years, and more than half of them had handled more than 10 postpartum cases over the previous two years prior to the study. The detail socio-demographic characteristics of respondents are shown in table 1.

**Table 1:** Traditional birth attendant demographics

Number	Pseudonym	Age	Marital status	Duration in Traditional birth attendance	Formal education	Number of mothers delivered and diagnosed with PPH in past two years	Sub-county and interview date.
**1**	TBA 1	44	Married	25 years	Yes	Less than 10	Bulamagi, July-17^th^-2020
**2**	TBA 2	53	Married	35 years	Yes	Less than 10	Nawandala, July-15th-2020
**3**	TBA 3	43	Married	24 years	Yes	More than 10	Kigulu South, July-17^th^-2020
**4**	TBA 4	44	Married	23 years	Yes	More than 10	Nabitende July-18-2020
**5**	TBA 5	56	Married	31 years	Yes	More than 10	Nakalama
**6**	TBA 6	57	Single (Separate)	27 years	No	Less than 10	Nakigo, July-16^th^-2020
**7**	TBA 7	56	Married	34 years	Yes	More than 10	Nakigo, July-16^th^-2020
**8**	TBA 8	70	Widow	38 years	No	More than 10	Nawandala, July-15^th^-2020

### Themes Generated from the Study

The predetermined themes and emergent themes are shown; the study had three predetermined themes, one being postpartum hemorrhage Assessment practices from which three sub-themes emerged. They included quantification of blood loss using household cups, vitals assessment and visual assessment. The second predetermined theme was resuscitation practices, from which three (3) emergent themes were obtained including warm water use, no resuscitation and intravenous infusion of fluids. The third predetermined theme was postpartum hemorrhage treatment practices from which four sub-themes emerged. These were herbal medicine use, referral, Uterine massage and the use of Uterotonics. Details are shown in table 2.

**Table 2:** Predetermined (A prior assumptions) and emergent themes

Predetermined theme	Emergent themes
**Postpartum hemorrhage Assessment practices**	Quantification of blood loss using household cups
	Vitals assessment
	Visual assessment
**Resuscitation practices**	Warm water use
	No resuscitation
	Intravenous infusion
**Postpartum hemorrhage treatment practices**	Herbal medicine use
	Referral
	Uterine massage
	Use of Uterotonics

### How traditional birth Attendants assess for postpartum hemorrhage

The exploration of postpartum hemorrhage assessment practices revealed that the traditional birth attendants carried out the assessment using three methods, that is; quantification of blood loss, vitals assessment and visual assessment. However, it is evident that visual assessment was the most dominant form of hemorrhage assessment that was used.

#### Sub-theme 1a: Quantification of blood loss using household cups

**Two of the traditional birth attendants mentioned** that they assessed blood loss, based on quantification in 500ml household cups. One of them, who had spent 25 years in traditional birth attendance, reported that it is when the blood lost from a woman after birth filled a cup then she ascertained postpartum hemorrhage. She mentioned that;

*“[…] When a woman gives birth, we have a quantity of blood that we base on to judge whether it is normal blood or it is postpartum hemorrhage” I personally base on whether the blood fills a tea cup or not, if it does, then I consider it to be hemorrhage […]”* TBA 1, 25 years in TBA practice, July-17th-2020

Another one, with 24 years of traditional birth attendance experience reported that she pours the blood in a basin and then estimates its quantity with a cup, which if filled, indicates postpartum hemorrhage. She mentioned that;

*“[…] At times I pour that blood into a basin, and I estimate the amount with a cup; if it fills the cup, I know it is postpartum hemorrhage, if it fills more than two cups, I immediately take the mother to Iganga hospital […]”* TBA 3, 24 years in TBA practice, July-17th-2020

Another TBA, with 31 years of experience in traditional birth attendance estimated blood loss using both table spoon measurements and household cup size, mentioning that normal blood loss does not exceed 10 table spoons, but that if the blood filled a cup, then it was postpartum hemorrhage.

*“[…] After I have delivered the mother, I take care to monitor the hemorrhage going on; usually normal hemorrhage after a vaginal birth is always little, like five to 10 table spoons, but if the blood exceeds the quantity that can fit in a normal household cup then I can tell that it is postpartum hemorrhage […]”* TBA 5, 31 years in TBA practice, July-18th-2020

#### Sub-theme 2a: Vitals assessment

Traditional birth attendants reported that they also assessed vitals (physiological makers), albeit using local methods. One of them mentioned is the use of physical appearance by assessing eye color and appearance as stated by TBA 2:

*“[…] The other assessments are related to physical assessments; mothers who hemorrhage a lot tend to look different, even the eyes become white and droopy […]”* TBA 2, 35 years in TBA practice, July-15th-2020

Assessment of physical appearance is also supplemented with the assessment of the rate of breathing as stated by another birth attendant who had almost a similar sentiment earlier reported by TBA 2.

*“[…] Sometimes you can check the eyes or breathing rate of the woman, to find out whether the woman has lost a lot of blood or not[…]”* TBA 8, 38 years in TBA practice, July-15th-2020

Another one reported that she assessed blood pressure and pulse by palpating some pressure points (around the wrist, neck region and the chest), with her justification for doing so being the lack of blood pressure measurement machines.

*“[…] I do not carry out any other assessment apart from the visual assessment of blood, although I usually touch some body parts to find out how fast the heart is beating, it does not show actually blood pressure since I have no machine to measure blood pressure […]”* TBA 1, 25 years in TBA practice, July-17th-2020

Another attendant, with 38 years of experience in traditional birth attendance out rightly opined that she does not assess any vitals.

*“[…] As traditional birth attendants we do not have machines for measuring blood pressure, so we do not carry out the assessment for some of those vitals […]”* TBA 8, 38 years in TBA practice, July-15th-2020

#### Sub-theme 3a: Visual assessment

Visual assessment of blood loss was the most common assessment method used, having been mentioned by all traditional birth attendants, some of whom combined it with vitals assessment, and quantification of blood lost using household cups. TBA 3, one of the attendants, with 24 years of experience in traditional birth attendance reported that she based on the color of blood to come to a conclusion that a woman was experiencing postpartum hemorrhage.

*“[…] It is easy to tell, blood lost during postpartum hemorrhage has a different color; it is usually bright red in color and when I see it, that is when I know that the women is experiencing post-partum hemorrhage […]”* TBA 3, 24 years in TBA practice, July-17th-2020

Similarly, TBA 7, with 34 years of experience in traditional birth attendance categorically mentioned that visual assessment was the main assessment method for post-partum hemorrhage. She explicitly mentioned that when the blood being hemorrhaged was light red in color, it is postpartum hemorrhage, and if it was dark red, it was normal hemorrhage normally associated with child birth.

*“[…] The main assessment is observing the amount of blood that the woman has lost, others like determining the heartbeat, how the woman is breathing and others are secondary […]”* TBA 7, 34 years in TBA practice, July-16th-2020

However, TBA 7 also mentioned that she based on the color of blood, reporting that:

*“[…] I also assess the hemorrhage based on the color of the blood, if the blood is light red, then I will know that it is postpartum hemorrhage, if the blood is dark red, then it is normal bleeding, that is not a causefor alarm […]”* TBA 7, 34 years in TBA practice, July-16th-2020

Similar sentiments were further shared by TBA 8, who reported that she also based on blood color to deduce whether the blood loss was normal or not;

*“[…] I can base on blood color; usually normal blood is darker in color, but if light red blood starts coming out, then I know that it is killer blood […]”* TBA 8, 38 years in TBA practice, July-15th-2020

TBA 7 also reported that on top of blood color, she based on how whether or not blood continued to be lost after cleaning with a cotton wool swab.

*“[…] For me I assess it visually, but using cotton wool, when the woman has given birth and she continues to bleed regardless, I clean that first blood with cotton wool, and when I do clean her more than twice and the blood continues to come, I get to know that it is postpartum hemorrhage […]”* TBA 7, 34 years in TBA practice, July-16-2020

TBA 6, with 27 years of experience in traditional birth attendance mentioned that she based her visual assessment on the rate of hemorrhage, stating that once the quantity of blood the woman was losing doubled in a few minutes, then it was postpartum hemorrhage that will have occurred.

*“[…] You can tell it is postpartum hemorrhage basing on how fast the blood is accumulating on the area in which the woman gave birth. If it is hemorrhage, you move out of the room for just 10 minutes, andyou will come back to find that the quantity of blood you left on the polythene sheet has doubled […]”* TBA 6, 27 years in TBA practice, July-16th-2020

TBA 2 on the other hand reported that, her diagnosis of postpartum hemorrhage is usually based on whether or not the blood lost makes the paddings drip blood.

*“[…] I assess postpartum hemorrhage using visual estimates; it is easy to notice blood that has exceeded normalcy. With the experience I have, I can tell the quantity of blood lost basing on the paddings I have put on the mother. They usually do not drip with blood even when soaked, but if they do, then that becomes postpartum hemorrhage […]”* TBA 2, 35 years in Traditional Birth practice, July-15th-2020

TBA 8, with 38 years' experience also had a similar assessment method used, on top of the blood color assessment, she had mentioned earlier. She said that;

*“[…] I carry out my assessment of postpartum hemorrhage basing on how soaked the paddings have put the mother on are. If the paddings get so much soaked, then I know it is postpartum hemorrhage […]”* TBA 8, 38 years in TBA practice, July-15th-2020

Five traditional birth attendants reported that they used visual assessments, but mostly based on the amount of blood that collected on the plastic (polythene) beddings that they delivered the mothers on. One of the attendants, TBA 1, with 25 years of experience reported that polythene sheet use made it easy to estimate blood loss

*“[…] Our mothers here give birth on plastic bag, so it is easy to tell when the bleeding is too much, since it will be collecting on the polythene bag […]”* TBA 1, 25 years in TBA practice, July-17th-2020

TBA 2 and TBA 6 had the same position regarding the use of polythene sheets, opining that;

*“[…] The assessment I usually carry out is usually visual, I can base on the amount of blood that has come out of the woman; that is easy to measure since we deliver them on plastic bags, the blood stays there and its quantity can easily be estimated […]”* TBA 2, 35 years in TBA experience, July-15-2020

TBA 6 shared that;

*“[…] It is easy to tell that the amount of blood lost is dangerous or not; after delivering the child, I keenly observe the amount of blood collecting on the plastic sheet on which the mother is lying. As the blood is collecting on the sheet, I can tell that it has exceed the normal amount or not […]”* TBA 6, 27 years in TBA practice, July-16-2020

TBA 3, with 24 years' experience, also reported that she visually based on the amount of blood that collected on the plastic sheet on which she delivered the mother. She mentioned that usually, mothers experiencing postpartum hemorrhage usually had more blood collected on the sheet than mothers having normal hemorrhage.

*“[…] I assess blood loss based on the amount of blood I find on the plastic polythene sheet on which I delivered the mother. It does not take much time, once it is postpartum hemorrhage, you will find the mother in something like a lake of blood […]”* TBA 3, 24 years in TBA practice, July-17-2020

TBA 4, with 23 years of experience reported that she also visually assessed the amount of blood lost, based on the number of blood pouches that formed on the plastic sheet. She said that;

*“[…] I estimate the blood loss visually of course, I do not have any machines or devices to measure blood quantity. When the blood forms many pouches on the plastic bag, that is when I know that it is life threatening […]”* TBA 4, 23 years in TBA practice, July-18th-2020

The resuscitation practices of mothers diagnosed with postpartum hemorrhage among traditional birth attendants

The exploration of resuscitation practices that traditional birth attendants used following the assessment and confirmation of postpartum hemorrhage mainly rotated around the use of warm water and intravenous infusion, which was only reported by one traditional birth attendant. However, by and large the traditional birth attendants sampled reported that they did not carry out any resuscitation.

#### Sub-theme 1b: Warm water and cloth use

Two traditional birth attendants reported that after ascertaining the incidence of postpartum hemorrhage they gave their patients warm drinks and warm pieces of cloth to support their health as they awaited further intervention. TBA 8, with 38 years of experience reported that she gave warm water to the mothers with postpartum hemorrhage, as she waited for transport to take the mother to the hospital

*“[…] In case of postpartum hemorrhage, I give the woman smasome warm water to sage herself, but if severe, then we take the mother to the hospital […]”* TBA 8, 38 years in TBA practice Attendance, July-15th-2020

She, however, quickly added that it was rare to give the warm water, and that it was only when a mother bled too much, at which point she also gave herbal medicine, in a bid to avoid escalation.

*“[…] I usually give them some warm water, but that is rare, once I notice that the woman has bled too much, I give her herbal medicine, before the situation gets out of hand […]”* TBA 8, 38 years in TBA practice, July-15th-2020

TBA 2 on the other hand reported that she sometimes gives the mothers a warm cloth, and requests them to massage themselves as they wait for transportation to the hospital.

*“[…]Sometimes I give them a warm cloth and ask them to massage their abdomen, as they wait for the motorcycle to come for them and take them to the hospital[…]”* TBA 2, 35 years in TBA practice, July-15th-2020

#### Sub-theme 2b: Intravenous infusion

One of the traditional birth attendants, TBA 3, with 24 years in traditional birth attendance mentioned that she went ahead to give intravenous infusions all in an effort to save the mother's life.

*“[…] Although we were given operational limits, for me I go ahead andput a cannula on the woman who has bled so much, out of the love to save her life. I put a drip on her and then call an ambulance to take her to the hospital […]”* TBA 3, 24 years in TBA practice, July-17th-2020

However, the assertion on intravenous infusion couldn't be verified by the investigators.

#### Sub-theme 3b: No resuscitation

It was however evident that most of the traditional birth attendants in Iganga do no resuscitate mothers they ascertain as experiencing postpartum hemorrhage. Most of those who reported that they do not resuscitate added that they always tried to avoid situations where mothers would be in very critical state.

TBA 1, with 25 years of experience reported that she could not conduct resuscitation because she had no facilities to do so but that she always ensured that she spent less than an hour with each mother who experiences postpartum hemorrhage

*“[…] We do not do a lot of resuscitation, because we do not have the means to, but then again, I personally spend less than an hour with a woman who has had postpartum hemorrhage, so I do not let her stay here for a long time to require resuscitation […]”* TBA 1, 25 years in TBA practice, July-17th-2020

TBA 1 added that;

*“[…] For me I do not wait for a situation to get worse, if the mother has had more than a cup of blood lost; I do not usually allow for resuscitation time, I call an ambulance or a motorcycle to take her to the hospital […]”* TBA 1, 25 years in TBA practice, July-17th-2020

With sentiments almost similar to those of TBA 1, TBA 8 also mentioned that;

*“[…] We do not have the means to resuscitate, and we do not need to, generally speaking, because we do not keep the mothers here for so long, that they lose too much blood to require resuscitating […]”* TBA 8, 38 years in TBA practice, July-15-2020

TBA 4 categorically stated that they were told not to carry out any resuscitation, during the training they had 20 years earlier

*“[…] During the training sessions we were given 20 years ago, we were told that we are not supposed to treat the mothers. So, for me I do not treat the mothers who experience postpartum hemorrhage. Once they hemorrhage, I send them to the health facility […]”* TBA 4, 23 years in TBA practice, July-18th-2020 Similarly, TBA 6 also reported that:

*“[…] I do not carry out any resuscitation, we are not allowed to, and besides, once I notice that it is postpartum hemorrhage going on, I do not wait for the mother to get into a health states that is so threatening to require resuscitation, I take her to the hospital or organize transportation to take her to the hospital […..]”* TBA 6, 27 years in TBA practice, July-16th-2020

TBA 7 also opined that she does not conduct any resuscitation because she always ensures that the mother she has delivered well and those who get PPH do not stay for a time frame that lets them get into critical conditions.

*“[…] I do not carry out any resuscitation, if I see that a lot of blood has been lost by the mother, I do not wait for them to slip into a situation that requires resuscitation, I refer them to the district hospital […]”* TBA 7,34 years in TBA practice, July-16th-2020

### Postpartum hemorrhage treatment interventions made by traditional birth attendants

Postpartum hemorrhage treatment interventions that were used by traditional birth attendants were four; the use of herbal medicine, the use of Uterotonics, uterine massage and lastly referral to hospital. Of the four treatment interventions, however, the use of herbal medicine (herbal Uterotonics), and referral to the hospital were the most prominent treatment interventions. This was followed by use of modern Uterotonics.

#### Sub-theme 1c: Herbal medicine use

Six of eight traditional birth attendants whose responses were considered in the analysis reported use of herbal Uterotonics as treatment for postpartum hemorrhage.

One of them, TBA 1, with 25 years of experience in traditional birth attendance, mentioned that as a Musoga traditional birth attendant, she had her own local and effective medicine she uses to stop bleeding among mothers with postpartum hemorrhage. She said;

*“[…] As a Musoga woman, I have my own medicine that I use to treat postpartum hemorrhage and stop bleeding; it is very effective in stopping the bleeding, but I will not tell you its name”. I use the bark of the tree which I grind in a mortar, and give the bleeding woman to drink, the bleeding stops immediately […]”* TBA 1, 25 years in TBA practice, July-17th-2020

TBA 8, with 35 years of experience mentioned that she also had her herbal medicines she used as Uterotonics, but was quick to add that she always sent the women to the hospital, irrespective of whether she had administered herbal Uterotonics to them or not.

*“[…] For me I have my herbal medicine that is effective is stopping bleeding- very effective, but I still send the mothers to the district hospital in case they bleed too much, not because the medicine is ineffective, but because I can't establish the cause of the hemorrhage. So, they go to the hospital I order to the cause to be established, if I could establish the cause, I could perhaps treat them from here […]”* TBA 8, 35 years in TBA practice, July-15th-2020

TBA 5, another traditional birth attendant, also mentioned that she gave herbal medicine after ascertaining that the mother had postpartum hemorrhage. She however added that she gave the herbal medicine regardless of what the causes of postpartum hemorrhage, but rather blood lost filling a cup

*“[…] I cannot tell whether the postpartum hemorrhage is due to failure of the uterus to contact, retained placenta, blood clotting irregularities or due to trauma, when the mother continues bleeding profusely and the blood quantity looks like it can fill a 250 ml cup, I give her my herbal medicine […]”* TBA 5, 31 years in TBA practice, July-18th-2020

The same was mentioned by TBA 2, of 35 years TBA experience, she mentioned that:

*“[…] I do not carry out any interventions to remove any retained placentae, in case it is the cause, I send the mother to a hospital, but still I give her my herbal medicine […]”* TBA 2, 35 years in TBA practice, July-15th-2020

However, there was one traditional birth attendant who mentioned that she does not give herbal medicines to any of the mothers who develop postpartum hemorrhage.

*“[…] I do not give them any medicine herbal or modern, once I ascertain that they have experienced postpartum hemorrhage, I call an ambulance or get a motorcycle to take them to the hospital […]”* TBA 4, 23 years in TBA practice, July-18th-2020

#### Sub-theme 2c: Use of modern Uterotonics

Three traditional birth attendants reported that they used modern uterotonics in the treatment of postpartum hemorrhage, although none of them practically administered the uterotonics. All of them mentioned that they had a nurse they liaised with to always come in and administer the drug in case it was needed.

*“[….] Some mothers come with their own oxytocin doses, and in case it is needed, I call a nurse to administer it […]”* TBA 1, 25 years in TBA practice, July-17th-2020

Another birth attendant, Aisha, mentioned that she used both herbal and modern uterotonics in case the modern uterotonics had not taken effect in preventing hemorrhage.

*“[…] There is a nurse that I work with in case a mother develops postpartum hemorrhage, she works at the district hospital; we give the mothers Pitocin, but in case the bleeding does not stop, I also use my own herbal medicine […]”* TBA 8, 38 years in TBA practice, July-15th-2020

TBA 3 also reported that she gave the mothers modern uterotonics, once she noticed continued bleeding after birth, and further mobilized transportation to the hospital in case the bleeding does not stop.

*“[…] When I diagnose postpartum hemorrhage, having seen bright red blood and profuse bleeding, I give herbal Uterotonics immediately and if the bleeding continues, I then call for transport to take the mother […]”* TBA 3, 25 years in TBA practice, July-17th-2020

#### Sub-theme 3c: Uterine massage

One traditional birth attendant mentioned that she sometimes carries out uterine massage with warm water, although at times she requested that the mothers should massage themselves.

*“[…] I can carry out uterine massage superficially, but at times, it is the mothers who do that themselves, I simply warm some water, and give them to massage themselves […]*” TBA 1, 25 years in TBA practice, July-17th-202

#### Sub-theme 4c: Referral

Referral of mothers with postpartum hemorrhage to the district hospital was widespread practice among the traditional birth attendants. All of them mentioned that in case they noticed that the hemorrhage would not stop after administration of modern uterotonics or herbal medicines, they sent the mothers to the hospital

TBA 4, for instance mentioned that:

*“[…] I do not give them any medicine herbal or modern, once I ascertain that they have experienced postpartum hemorrhage, I call an ambulance or get a motorcycle to take them to the hospital […]”* TBA 4, 23 years in TBA practice, July-18th-2020

TBA 5, however, mentioned that she calls in an ambulance only when she notices that the mother is getting unconscious. She mentioned that;

*“[…] For me when I notice that the woman's health status is deteriorating, when she starts getting unconscious, I immediately call a motorcycle to take her to the hospital. If the woman has her relatives around who have cars, they take her to the hospital […]”* TBA 5, 31 years in TBA practice, July-18th-2020

TBA 8 reported that she considered sending a mother to the hospital, if the mother experienced refractory postpartum hemorrhage.

*“[…] For rebellious PPH, the only solution remains to send the woman to the hospital; if I give the herbal medicine first and the bleeding does not stop, I call the nurse and we give Pitocin, if the bleeding does not stop, then we send the woman to the hospital immediately”. I usually spend less than an hour with a woman who has bled[…]”* TBA 8, 38 years in TBA practice, July-15th-2020

A similar sentiment was shared by TBA 3, with 25 years of TBA experience; she mentioned that whereas she gave the mothers herbal medicine, she still gave them referral notes to the hospital. She mentioned that;

*“[…] However, even after me giving the woman that herbal medicine of mine, I still write a referral note to the main hospital in Nakavule – Iganga hospital[…]”* TBA 3, 25 years in TBA practice, July-17th-2020

## Discussion

### Postpartum hemorrhage assessment practices of traditional birth attendants

The findings showed that the TBAs carried out assessment of PPH using three methods; quantification of blood lost using household cup size, vitals assessment and visual assessment. These findings are consistent with other studies that have assessed PPH management practices in which visual and quantification methods of blood loss assessment were used^31^–^35^. The only difference is in quantification, where respondents in this study used rudimentary collection items (Cups), as opposed to the modern ones such as Collector Bag and novel birthing drapes reported in other studies. Again, this current study found that visual assessment was the most dominant form of hemorrhage assessment, which is consistent with findings by Prata, Bell, Holston & Quaiyum^36^ and Afshari, Soltani, Abedi & Kianfar^37^. It was somewhat dissimilar to findings by Bell, et al.^34^, Ochen, et al.^33^ and Chen, et al.^38^, in which quantification was the most commonly used practice. The difference in the findings is because the study by Bell, et al.^34^ was conducted immediately after a training had been offered to the respondents, resuling into better practice. The other study by Ochen, et al.^33^ was conducted among attendants who had access to modern quantification tools including gauzes and blood discs, which made them adopt quantification, as compared to this current study. The finding that visual assessments were more wide spread among TBAs in Iganga is of great concern given the fact that visual estimation of blood loss has been established as being the most inaccurate assessment method^31^,^32^,^39^. This gap in assessment could increase the risk of a mother experiencing a near miss^11^. With visual assessment, the establishment of the 500ml threshold, which is indicative of PPH, can be very challenging. This was evident among TBAs in Iganga district, some of whom delivered mothers on pieces of cloth, only gauging blood loss by how soaked the beddings are.

### Resuscitation practices of mothers diagnosed with postpartum hemorrhage among traditional birth attendants

Resuscitation of mothers diagnosed with PPH is done for purposes of restoring blood volume^8^, and augmenting the capacity of the blood to carry oxygen^40^. This process involves the use of intravenous solutions^8^,^40^,^41^ and oxygen therapy^41^, all of which cannot be carried out by TBAs. However, this study found that traditional birth attendants did not carry out any resuscitation. On a positive note, the TBAs only applied basic resuscitation procedures including the provision of warm water to the mothers, which was within their practice right and realm, associated with no side effects. However, only one traditional birth attendant reportedly provided intravenous solutions, which although consistent with some studies^35^,^42^, is contraindicated among TBAs^40^. Majority of the TBAs in Iganga did not resuscitate mothers experiencing postpartum hemorrhage. They reported that they always endeavor to avoid situations that would require resuscitation.

### Postpartum hemorrhage treatment interventions made by traditional birth attendants

Consistent with standard practice, traditional birth attendants in Iganga were found to have a number of treatment interventions they initiate following PPH assessment and diagnosis. This is completely contrary for findings in many other studies^43^–^47^. However, the treatment intervention that was used in this current study was herbal. This difference (treatment intervention practices) is expected, since by law, traditional birth attendants are not supported to carry out uterine massage, administer modern uterotonics, or carry out any surgical interventions.

### Use of Herbal uterotonics and their impact on maternal outcomes

From this study, it is clear that the TBAs have good degree of information on use of herbal uterotonic to achieve good maternal outcomes. However, they have very limited information on the impact on maternal outcomes. This is very dangerous and can lead to maternal death or fetal death or both. A related study in Uganda found that the prevalence of herbal medicines use during pregnancy was high at 20%, and was commonly used in the second and third 21 % trimesters. In Ghana, it was found that the use of herbal uterotonics is common among TBAs with varying impacts^48^. This is similar in many other studies^49^-^51^.

## Recommendations

The authors recommend as follows;

1) The ministry of health and the global stakeholders should prioritize sensitization campaigns through radio talk-shows and other media to increase awareness about health facility deliveries
2) Ministry of health should plan and device positive incentives for traditional birth attendants who refer mothers to hospitals for delivery by skilled birth attendants
3) The district health office together with the ministry of health and the global stakeholders should consider community dialogues with tradition birth attendants and the general community to enhance referral of mothers for hospital delivery.
4) At the family level, both locally in Uganda and across the globe, couples are recommended to discuss and plan their deliveries early enough as soon as conception occurs.
5) At the health facility level, midwives and doctors are recommended to adopt positive attitudes toward mothers who come to deliver in health facilities

### Study implication and limitations

This study implies that encouraging TBAs to refer mothers for skilled birth attendance at health facilities could provide positive synergies to improve health facility delivery. This is especially when they are given sufficient incentives to refer as soon as possible. However, it should be noted that this study was qualitative and carried out in only one urban setting and thus findings may not be generalized to a much larger population. More studies are, therefore, called in this field of study.

## Conclusion

TBAs in Iganga district visually assess postpartum hemorrhage, do not conduct some form of resuscitation procedures and use herbal uterotonics in the treatment of postpartum hemorrhage alongside with referral to hospital. As traditional birth attendants remain outlawed in Uganda, it is important that they are encourage to refer mothers to hospitals for proper management of PPH. Since significant number of childbirths are attended to by TBAs, providing them with positive incentives for referral would make them actively engage in referral and improve skilled delivery. Gradual engagement in this process to improve the main stream health system should be optimized, as this will complement the activities of skilled birth attendants, and perhaps ease the burden on maternal health care.

## Data Availability

The data related to this study is available with principal investigator and can be availed with reasonable request.

## APPENDIX: IN-DEPTH INTERVIEW GUIDE

1) How do you usually carry out assessments of mothers you suspect to have experienced postpartum hemorrhage during birth? What assessments do you usually consider most [Interviewer probes for: ongoing assessment of blood loss, Visual assessments of blood loss, Weighing all blood loss, Also consider clinical signs and symptoms of hypovolemia, unconsciousness, fainting, speed of blood flow, the woman's prior hemoglobin] …………………………………………………………………………………………Do you usually involve any other assistant in cases when postpartum hemorrhage is suspected? If yes, who do you involve and what role do they usually play in the process? [Interviewer probes for: recording of events, documentation of assessments findings, vital signs]…………………………………………………………………………….
2) What vital signs do you usually look out for whilst carrying out the assessments for postpartum hemorrhage? [Interviewer probes for: brisk bleeding, pulse rising (rate), blood pressure falling, maternal symptoms of hypotension, heart rate, breathing rate, uterine size and tenderness, pain/cramping, vaginal loss] …………………………………………………………………………………………
3) Tell me about what you usually do next in cases when a mother displays all the signs of postpartum hemorrhage we talked about earlier [Interviewer probe for: resuscitation and its associated practices including calling for help, performance of uterine massage, any fluids administered (route of administration), or any other resuscitation practice at this point, keeping patient warm, monitoring of blood pressure, monitoring of urine output, consideration of lab test] …………………………………………………………………………………………
4) Please elaborate on how you carry out any of the interventions you have mentioned above (in case the attendant mentioned any) [Interviewer probe for practical procedures to that effect] …………………………………………………………………………………………
5) Do you carry out any other investigations at this stage when you have already confirmed postpartum hemorrhage? If yes, which ones [Interviewer probe for: assessment of signs of anemia, including fatigue, shortness of breath, chest pain, lactation problems]? …………………………………………………………………………………………
6) Do you make any treatment or non-treatment related interventions meant to stop bleeding, for mothers who have had postpartum hemorrhage? If yes, how do you attempt to treat a mother whose uterus has not contracted (atony or tone) [Interviewer probes for: massaging of the atonic uterus to stimulate contraction and expel clots, administration of oxytocics, and insertion of indwelling catheter (IDC] …………………………………………………………………………………………
7) How do you attempt to treat a mother who has trauma [Interviewer probes for: checking of the genital tract for lacerations, and haematomas to exclude bleeding from trauma, application of pressure, in case of visible trauma, vaginal or uterine packing]? …………………………………………………………………………………………
8) How do you attempt to treat a mother who has had a retained placenta [Interviewer probes for: inspection of placenta, exploration of uterus, manual removal of placenta, checking placenta for completeness, if delivered]? …………………………………………………………………………………………
9) How do you attempt to treat a mother whose blood has not clotted, following a full hour of bleeding?……………………………………………………………………………………………………….
10) Are there any other postpartum hemorrhage management practices that you carry out, but haven't shared with me yet? If so, would like to share with me? …………………………………………………………………………………………
